# Can machine-learning improve cardiovascular risk prediction using routine clinical data?

**DOI:** 10.1371/journal.pone.0174944

**Published:** 2017-04-04

**Authors:** Stephen F. Weng, Jenna Reps, Joe Kai, Jonathan M. Garibaldi, Nadeem Qureshi

**Affiliations:** 1 NIHR School for Primary Care Research, University of Nottingham, Nottingham, United Kingdom; 2 Division of Primary Care, School of Medicine, University of Nottingham, Nottingham, United Kingdom; 3 Advanced Data Analysis Centre, University of Nottingham, Nottingham, United Kingdom; 4 School of Computer Science, University of Nottingham, Nottingham, United Kingdom; Harbin Institute of Technology Shenzhen Graduate School, CHINA

## Abstract

**Background:**

Current approaches to predict cardiovascular risk fail to identify many people who would benefit from preventive treatment, while others receive unnecessary intervention. Machine-learning offers opportunity to improve accuracy by exploiting complex interactions between risk factors. We assessed whether machine-learning can improve cardiovascular risk prediction.

**Methods:**

Prospective cohort study using routine clinical data of 378,256 patients from UK family practices, free from cardiovascular disease at outset. Four machine-learning algorithms (random forest, logistic regression, gradient boosting machines, neural networks) were compared to an established algorithm (American College of Cardiology guidelines) to predict first cardiovascular event over 10-years. Predictive accuracy was assessed by area under the ‘receiver operating curve’ (AUC); and sensitivity, specificity, positive predictive value (PPV), negative predictive value (NPV) to predict 7.5% cardiovascular risk (threshold for initiating statins).

**Findings:**

24,970 incident cardiovascular events (6.6%) occurred. Compared to the established risk prediction algorithm (AUC 0.728, 95% CI 0.723–0.735), machine-learning algorithms improved prediction: random forest +1.7% (AUC 0.745, 95% CI 0.739–0.750), logistic regression +3.2% (AUC 0.760, 95% CI 0.755–0.766), gradient boosting +3.3% (AUC 0.761, 95% CI 0.755–0.766), neural networks +3.6% (AUC 0.764, 95% CI 0.759–0.769). The highest achieving (neural networks) algorithm predicted 4,998/7,404 cases (sensitivity 67.5%, PPV 18.4%) and 53,458/75,585 non-cases (specificity 70.7%, NPV 95.7%), correctly predicting 355 (+7.6%) more patients who developed cardiovascular disease compared to the established algorithm.

**Conclusions:**

Machine-learning significantly improves accuracy of cardiovascular risk prediction, increasing the number of patients identified who could benefit from preventive treatment, while avoiding unnecessary treatment of others.

## Introduction

Globally, cardiovascular disease (CVD) is the leading cause of morbidity and mortality. In 2012, there were 17.5 million deaths from CVD with 7.4 million deaths due to coronary heart disease (CHD) and 6.7 million deaths due to stroke [1]. Established approaches to CVD risk assessment, such as that recommended by the American Heart Association/American College of Cardiology (ACC/AHA), predict future risk of CVD based on well-established risk factors such as hypertension, cholesterol, age, smoking, and diabetes. These risk factors have recognised aetiological associations with CVD and feature within most CVD risk prediction tools (e.g. ACC/AHA [2], QRISK2 [3], Framingham [4], Reynolds [5]. There remain a large number of individuals at risk of CVD who fail to be identified by these tools, while some individuals not at risk are given preventive treatment unnecessarily. For instance, approximately half of myocardial infractions (MIs) and strokes will occur in people who are not predicted to be at risk of cardiovascular disease [6].

All standard CVD risk assessment models make an implicit assumption that each risk factor is related in a linear fashion to CVD outcomes [7]. Such models may thus oversimplify complex relationships which include large numbers of risk factors with non-linear interactions. Approaches that better incorporate multiple risk factors, and determine more nuanced relationships between risk factors and outcomes need to be explored.

Machine-learning (ML) offers an alternative approach to standard prediction modelling that may address current limitations. It has potential to transform medicine by better exploiting ‘big data’ for algorithm development [7]. ML developed from the study of pattern recognition and computational learning (so-called ‘artificial intelligence’). This relies on a computer to learn all complex and non-linear interactions between variables by minimising the error between predicted and observed outcomes [8]. In addition to potentially improving prediction, ML may identify latent variables, which are unlikely to be observed but might be inferred from other variables [9].

To date, there has been no large-scale investigation applying machine-learning for prognostic assessment in the general population, using routine clinical data. The aim of this study was to evaluate whether machine-learning can improve accuracy of cardiovascular risk prediction within a large general primary care population. We also sought to determine which class of machine-learning algorithm has highest predictive accuracy.

## Methods

### Data source

The cohort of patients was derived from the Clinical Practice Research Datalink (CPRD), anonymized electronic medical records from nearly 700 UK family practices documenting demographic details, history of medical conditions, prescription drugs, acute medical outcomes, referrals to specialists, admissions to hospitals, and biological results. The database is representative of the UK general population and linked to hospital (secondary care) records [10]. Ethical and research approvals were granted by the Independent Scientific Advisory Committee (ISAC) at CPRD (number 14_205).

### Study population

The cohort of patients were registered with a family practice between the ages of 30 to 84 years at baseline, who had complete data for the eight core baseline variables (gender, age, smoking status, systolic blood pressure, blood pressure treatment, total cholesterol, HDL cholesterol, and diabetes) used in the established ACC/AHA 10-year risk prediction model [2]. The baseline date was set as the 1^st^ of January 2005, thus allowing all patients within the cohort to be followed-up for 10 years. The end of the study period was specified as the 1^st^ of January 2015, the latest date for which CPRD had provided an updated dataset. Individuals with a previous history of CVD, lipid disorders which are inherited, prescribed lipid lowering drugs, or outside the specified age range prior to or on the baseline date were excluded from the analysis.

### Risk factor variables

The eight core risk variables (above) were used to derive a baseline risk prediction model using the published equations in the 2013 ACC/AHA guidelines for assessment of CVD risk [2]. To compare the machine-learning algorithms, an additional 22 variables with potential to be associated with CVD were included in the analysis. These variables were selected based on their inclusion in published CVD risk algorithms [2–5], within literature on other potential CVD risk factors [11–21], and further reviewed by practising clinicians (NQ, JK).

In nine of the additional continuous variables, there were some levels of missing data. Median imputation, a common approach to dealing with missing values in machine-learning algorithms [22] was used. It was also hypothesized that missing values in certain clinical variables (e.g. BMI and laboratory results) may indicate a perception of reduced relevance in certain patients, given the under recording of normal BMI values in primary care medical records [23]. Dummy variables were created to indicate whether these continuous variable values were missing. For demographic categorical variables, Townsend deprivation index (28) and ethnicity, missing values were given a separate category of ‘unknown’ in the analyses. In total, there were 30 variables (excluding dummy variables for missing values) analysed in the machine-learning models prior to baseline (**Table 1**).

**Table 1:** Variables included in the machine-learning algorithms.

Variable	Description	Reference^+^
Gender	male/female	[2–5]
Age	Years	[2–5]
Total cholesterol	mmol/L	[2–5]
HDL cholesterol	mmol/L	[2–5]
Systolic blood pressure	mm HG	[2–5]
Blood pressure treatment (anti-hypertensives prescribed)	yes/no	[2–4]
Smoking	yes/no	[2–5]
Diabetes	yes/no	[2–4]
Body mass index (BMI)	kg/m^2^	[3,4]
LDL cholesterol	mmol/L	[24]
Triglycerides	mmol/L	[24]
C-reactive protein (CRP)	mg/L	[5]
Serum fibrinogen	g/L	[12]
Gamma glutamyl transferase (gamma GT)	IU/L	[14]
Serum creatinine	g/L	[20]
Glycated haemoglobin (HbA1c)	%	[11]
Forced Expiratory Volume (FEV1)	%	[18]
AST/ALT ratio	—	[21]
Family history of CHD < 60 years	yes/no	[3,5]
Ethnicity	White Caucasian; South Asian; Black/Afro-Carribean; Chinese/East Asian; Other/Mixed; Unknown	[3]
Townsend deprivation index*	1^st^ quintile (most affluent)– 5^th^ quintile (most deprived); unknown	[3]
Hypertension	yes/no	[2–4]
Rheumatoid arthritis	yes/no	[3]
Chronic kidney disease	yes/no	[3]
Atrial fibrillation	yes/no	[3]
Chronic obstructive pulmonary disease (COPD)	yes/no	[15]
Severe mental illness	yes/no	[16]
Prescribed anti-psychotic drug	yes/no	[17]
Prescribed oral corticosteroids	yes/no	[19]
Prescribed immunosuppressant	yes/no	[13]

* Measures area level deprivation in the population based on unemployment, non-car ownership, non-home ownership, and household overcrowding

^+^ Inclusion in published cardiovascular risk algorithms or literature on other potential cardiovascular risk factors

### Outcome

The primary outcome was the first recorded diagnosis of a fatal or non-fatal cardiovascular event documented in the patient’s primary or secondary care computerised record. In primary care, CVD is labelled and electronically recorded by UK National Health Service (NHS) Read codes. Further, confirmation of outcomes in secondary care (Hospital Episodes Statistics) utilised ICD-10 codes, specifically I20 to I25 for coronary (ischaemic) heart conditions and I60 to I69 for cerebrovascular conditions.

### Machine-learning algorithms

To compare machine-learning risk algorithms, the study population was split in the data set into a ‘training’ cohort in which the CVD risk algorithms were derived and a ‘validation’ cohort in which the algorithms were applied and tested. The ‘training’ cohort was derived from random sampling of 75% of the extracted CPRD cohort, and the ‘validation’ cohort comprised the remaining 25%. Four commonly used classes of machine-learning algorithms were utilised: logistic regression [25], random forest [26], gradient boosting machines [27], and neural networks [28]. These algorithms were selected based on the ease of implementation into current UK primary care electronic health records. Development of the risk algorithms in the training cohort and application of the risk algorithms to the validation cohort was completed using RStudio with library packages *caret* (http://CRAN.R-project.org/package=caret) for neural networks and *h2o* (http://www.h2o.ai) for the remaining algorithms. Each model’s hyper parameters were determined by using a grid search and two fold cross-validation on the training cohort to determine the values which led to the best performance. Further details on machine-learning models are described in the **S1 Text.**

### Statistical analysis

Descriptive characteristic of the study population were provided, including number (%) and mean (SD) for categorical and continuous variables, respectively. The performance of the machine-learning prediction algorithms, developed from the training cohort, was assessed using the validation cohort by calculating Harrell’s c-statistic [29], a measure of the total area under the receiver operating characteristic curve (AUC). Standard errors and 95% confidence intervals were estimated for the c-statistic using a jack-knife procedure [30]. Additionally, using thresholds corresponding to the 10-year CVD risk of > 7.5% as recommended by the ACC/AHA guidelines [2] for initiating lipid lowering therapy, binary classification analysis was used to compare observed and expected prediction of cases and non-cases in the validation cohort. This process provided sensitivity, specificity, positive predictive value (PPV), and negative predictive value (NPV). The statistical analyses assessing algorithm performance were performed using STATA 13 MP4.

## Results

### Data extraction

**Fig 1:**
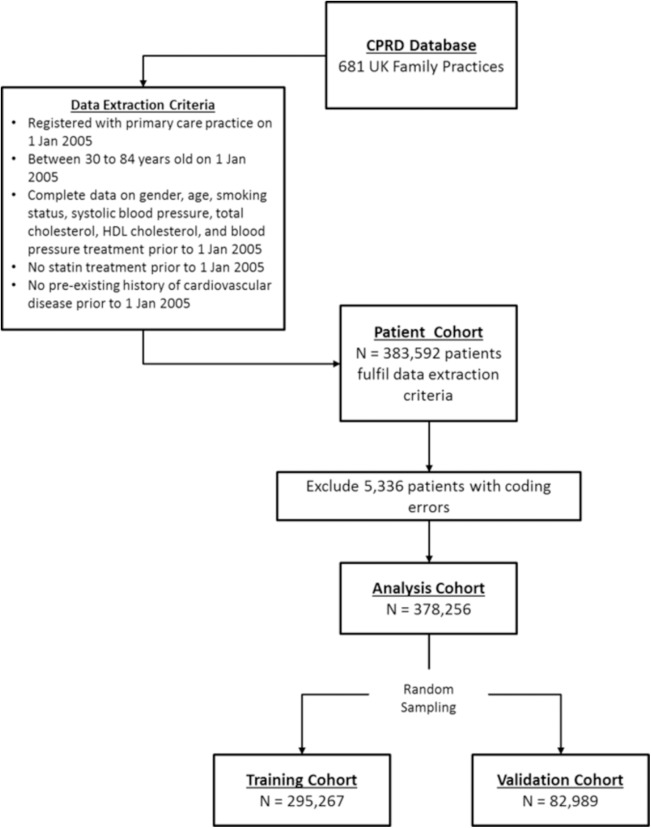
Patient cohort data extraction procedures.

There were a total of 383,592 patients from 12 million patients in the CPRD database at baseline (1 Jan 2005) who met eligibility criteria. After excluding 5,336 patients with coding errors (i.e. non-numerical entries for blood pressure/cholesterol) and extreme outlying observations (> 5 SDs from the mean), the analysis cohort consisted of 378,256 patients. This cohort was then randomly split into a 75% sample of 295,267 patients to train the machine-learning algorithms and the remaining sample of 82,989 patients for validation (**Fig 1**).

### Study population characteristics

From a total cohort of 378,256 patients who were free from CVD at baseline, there were 24,970 incident cases (6.6%) of CVD during the 10-year follow-up period. There were significantly fewer women than men (42% F, 52% M) in CVD cases while there was only slightly more women than men in non-CVD cases (52% F, 48% M). The mean baseline age of CVD patients was 65.3 years compared to 57.3 years in non-CVD patients (p < 0.001). Further characteristics of CVD and non-CVD patients are presented in **Table 2.**

**Table 2:** Characteristics of patients aged 30 to 84 in the CPRD study cohort who were free from CVD at baseline. Patients are stratified by first CVD event during the 10-year follow-up period.

Risk Factor Variables	Units	CVD (n = 24,970)	No CVD (n = 353,286)	P-Value
Age*	years (SD)	65.3 (11.1)	57.6 (12.8)	< 0.001
BMI^+^	kg/m^2 (SD)	27.9 (4.94)	27.9 (5.21)	0.323
Systolic blood pressure*	mm HG (SD)	141 (17.6)	137 (17.2)	< 0.001
Total cholesterol*	mmol/L (SD)	5.60 (1.11)	5.56 (1.06)	< 0.001
HDL cholesterol*	mmol/L (SD)	1.39 (0.41)	1.46 (0.43)	< 0.001
LDL cholesterol	mmol/L (SD)	3.45 (0.91)	3.40 (0.88)	< 0.001
Triglycerides^+^	mmol/L (SD)	1.69 (0.85)	1.57 (0.83)	< 0.001
CRP^+^	mg/L (SD)	10.0 (13.7)	8.37 (11.5)	< 0.001
Serum fibrinogen^+^	g/L (SD)	3.86 (1.22)	3.73 (1.33)	0.129
gamma GT^+^	IU/L (SD)	41.3 (33.7)	39.3 (33.6)	< 0.001
Serum creatinine^+^	umol/L (SD)	91.9 (17.3)	87.6 (16.0)	< 0.001
HbA1c^+^	% (SD)	7.26 (1.61)	7.14 (1.64)	< 0.001
FEV1^+^	% (SD)	66.2 (16.3)	67.8 (16.9)	0.007
AST/ALT ratio^+^	— (SD)	1.04 (0.36)	1.01 (0.35)	< 0.001
Female*	%	41.8	52.8	< 0.001
Smoking*	%	23.4	20.5	< 0.001
Family history CHD < 60 years	%	5.00	5.51	< 0.001
Ethnicity^a^: South Asian	%	2.27	1.90	0.004
Ethnicity^a^: Black/Afro-Caribbean	%	0.66	1.20	< 0.001
Ethnicity^a^: Chinese/East Asian	%	0.54	0.58	0.465
Ethnicity^a^: Other/Mixed	%	0.85	1.32	< 0.001
Ethnicity^a^: Unknown	%	43.5	57.1	< 0.001
SES^b^: 2nd Townsend quintile	%	15.8	16.0	< 0.001
SES^b^: 3rd Townsend quintile	%	13.7	13.6	< 0.001
SES^b^: 4th Townsend quintile	%	12.6	11.8	< 0.001
SES^b^: 5th Townsend quintile (most deprived)	%	7.95	6.91	< 0.001
SES^b^: Unknown	%	34.6	34.5	< 0.001
Hypertension	%	31.8	25.2	< 0.001
Diabetes	%	15.0	10.1	< 0.001
Blood pressure treatment*	%	28.3	21.9	< 0.001
Rheumatoid arthritis	%	1.55	0.91	< 0.001
Chronic kidney disease	%	0.99	0.48	< 0.001
Atrial fibrillation	%	4.64	2.20	< 0.001
COPD	%	3.97	2.02	< 0.001
Severe mental illness	%	0.34	0.32	0.563
Anti-psychotic drug prescribed	%	15.2	12.7	< 0.001
Oral corticosteroid prescribed	%	13.2	9.55	< 0.001
Immunosuppressant prescribed	%	13.3	9.70	< 0.001
BMI missing	%	3.48	5.87	< 0.001
LDL cholesterol missing	%	25.1	24.6	0.041
Triglycerides missing	%	11.7	12.3	0.004
CRP missing	%	88.5	89.9	< 0.001
Serum fibrinogen missing	%	99.0	99.0	0.207
gamma GT missing	%	64.8	69.1	< 0.001
Serum creatinine missing	%	16.1	21.5	< 0.001
HbA1c missing	%	79.6	85.9	< 0.001
FEV1 missing	%	96.3	97.7	< 0.001
AST/ALT ratio missing	%	85.2	88.2	< 0.001

*core risk factor for ACC/AHA 10-year CVD risk equations

^+^missing values present

^a^reference category is White Caucasian

^b^reference category is 1st Townsend quintile (most affluent)

### Machine-learning variable rankings

All variables listed in **Table 2**were inputs for the machine-learning models and trained using a cohort of 295,267 patients with 19,487 incident CVD cases (6.6%) of developing over the 10-year follow-up period. Variable importance was determined by the coefficient effect size for the ACC/AHA baseline model and machine-learning logistic regression. Random forest and gradient boosting machine models, based on decision-trees, rank variable importance on the selection frequency of the variable as a decision node while neural networks use overall weighting of the variable within the model. The top 10 risk factors for the CVD prediction algorithms are presented in **Table 3**.

**Table 3:** Top 10 risk factor variables for CVD algorithms listed in descending order of coefficient effect size (ACC/AHA; logistic regression), weighting (neural networks), or selection frequency (random forest, gradient boosting machines). Algorithms were derived from training cohort of 295,267 patients.

ACC/AHA Algorithm		Machine-learning Algorithms			
Men	Women	ML: Logistic Regression	ML: Random Forest	ML: Gradient Boosting Machines	ML: Neural Networks
Age	Age	Ethnicity	Age	Age	Atrial Fibrillation
Total Cholesterol	HDL Cholesterol	Age	Gender	Gender	Ethnicity
*HDL Cholesterol*	Total Cholesterol	SES: Townsend Deprivation Index	Ethnicity	Ethnicity	Oral Corticosteroid Prescribed
Smoking	Smoking	Gender	Smoking	Smoking	Age
Age x Total Cholesterol	Age x *HDL Cholesterol*	Smoking	*HDL cholesterol*	*HDL cholesterol*	Severe Mental Illness
Treated Systolic Blood Pressure	Age x Total Cholesterol	Atrial Fibrillation	HbA1c	Triglycerides	SES: Townsend Deprivation Index
Age x Smoking	Treated Systolic Blood Pressure	Chronic Kidney Disease	Triglycerides	Total Cholesterol	Chronic Kidney Disease
Age x *HDL Cholesterol*	Untreated Systolic Blood Pressure	Rheumatoid Arthritis	SES: Townsend Deprivation Index	HbA1c	*BMI missing*
Untreated Systolic Blood Pressure	Age x Smoking	Family history of premature CHD	BMI	Systolic Blood Pressure	Smoking
Diabetes	Diabetes	COPD	Total Cholesterol	SES: Townsend Deprivation Index	Gender

Italics: Protective Factors

The standard risk factors in the ACC/AHA algorithm stratified by gender were age, total cholesterol, HDL cholesterol, smoking, blood pressure, and diabetes. Several of these risk factors in the ACC/AHA model (age, gender, smoking) were present as top ranked risk factors for all four machine-learning algorithms. However, diabetes, which is prominent in many CVD algorithms, was not present in the top ranked risk factors for any of the machine-learning models (though HbA1c was included as a proxy in random forest models). Other new risk factors not found in any previous risk prediction tools but determined by machine-learning included medical conditions such as COPD and severe mental illness, prescribing of oral corticosteroids, as well as biomarkers such as triglyceride levels. Random forest and gradient boosting machines were most similar in risk factor selection and rankings, with some discrepancies in ranking order and substitution of BMI for systolic blood pressure. Logistic regression and neural networks prioritised medical conditions such as atrial fibrillation, chronic kidney disease, and rheumatoid arthritis over biometric risk factors. Neural networks also put less weighting on age as a risk factor, and included ‘*BMI missing*’ as a protective risk factor of CVD. Full variable selection rankings can be found in **S1 Table**.

### Prediction accuracy

The prediction accuracy according to the discrimination (AUC *c-*statistic) is shown in **Table 4**for all models

**Table 4:** Performance of the machine-learning (ML) algorithms predicting 10-year cardiovascular disease (CVD) risk derived from applying training algorithms on the validation cohort of 82,989 patients. Higher c-statistics results in better algorithm discrimination. The baseline (BL) ACC/AHA 10-year risk prediction algorithm is provided for comparative purposes.

Algorithms	AUC c-statistic	Standard Error*	95% Confidence Interval		Absolute Change from Baseline
			LCL	UCL	
BL: ACC/AHA	0.728	0.002	0.723	0.735	—
ML: Random Forest	0.745	0.003	0.739	0.750	+1.7%
ML: Logistic Regression	0.760	0.003	0.755	0.766	+3.2%
ML: Gradient Boosting Machines	0.761	0.002	0.755	0.766	+3.3%
ML: Neural Networks	0.764	0.002	0.759	0.769	+3.6%

*Standard error estimated by jack-knife procedure [30]

The ACC/AHA risk model served as a baseline for comparison (AUC 0.728, 95% CI 0.723–0.735). All machine-learning algorithms tested achieved statistically significant improvements in discrimination compared to the baseline models (from 1.7% for random forest algorithms to 3.6% for neural networks)

### Classification analysis

The ACC/AHA baseline model predicted 4,643 cases correctly from 7,404 total cases, resulting in a sensitivity of 62.7% and PPV of 17.1%. The random forest algorithm resulted in a net increase of 191 CVD cases from the baseline model, increasing the sensitivity to 65.3% and PPV to 17.8% while logistic regression resulted in a net increase of 324 CVD cases (sensitivity 67.1%; PPV 18.3%). Gradient boosting machines and neural networks performed best, resulting in a net increase of 354 (sensitivity 67.5%; PPV 18.4%) and 355 CVD (sensitivity 67.5%; PPV 18.4%) cases correctly predicted, respectively.

The ACC/AHA baseline model correctly predicted 53,106 non-cases from 75,585 total non-cases, resulting in a specificity of 70.3% and NPV of 95.1%. The net increase in non-cases correctly predicted compared to the baseline ACC/AHA model ranged from 191 non-cases for the random forest algorithm to 355 non-cases for the neural networks. Full details on classification analysis can be found in **S2 Table**.

## Discussion

Compared to an established AHA/ACC risk prediction algorithm, we found all machine-learning algorithms tested were better at identifying individuals who will develop CVD and those that will not. Unlike established approaches to risk prediction, the machine-learning methods used were not limited to a small set of risk factors, and incorporated more pre-existing medical conditions. Neural networks performed the best, with predictive accuracy improving by 3.6%. This is an encouraging step forward. For example, the addition of emerging biochemical risk factors, such as high sensitivity C-reactive protein, has recently achieved less than 1% improvement in CVD risk prediction [31].

### Strengths

To our knowledge, this is the first investigation applying machine-learning to routine data in patients’ electronic records, demonstrating improved prediction of CVD risk in a large general population. The study also illustrates use of a range of machine learning methods, as well as evaluation techniques, that are lacking in existing applications of machine-learning to clinical data [32]. Our results are consistent with much smaller studies [33,34] in more selected populations. For example, a cohort study of 5,159 men in Northern Germany [34] found a similar 3.2% improvement in accuracy of prediction of coronary risk using a probabilistic neural network model.

The current study’s use of an array of machine-learning algorithms has suggested intriguing variations in the importance of different risk factors depending on the modelling technique. Models based on decision trees resembled closely to each other, with gradient boosting machines out-performing random forests. Neural networks and logistic regression placed far more importance on categorical variables and CVD-associated medical conditions, clustering patients with similar characteristics in each groups. This may help inform further exploration of diverse predictive risk factors, and future development of new risk prediction approaches and algorithms.

Finally, the importance of missing values or non-response are not often assessed in development of conventional CVD risk prediction tools [2–5]. This study suggests that missing values, in particular, for routine biometric variables such as BMI, are independent predictors of CVD. This is consistent with subjective assessment by clinicians who may not record normal BMI values if patients appear at lower CVD risk [23].

### Limitations

**Fig 2:**
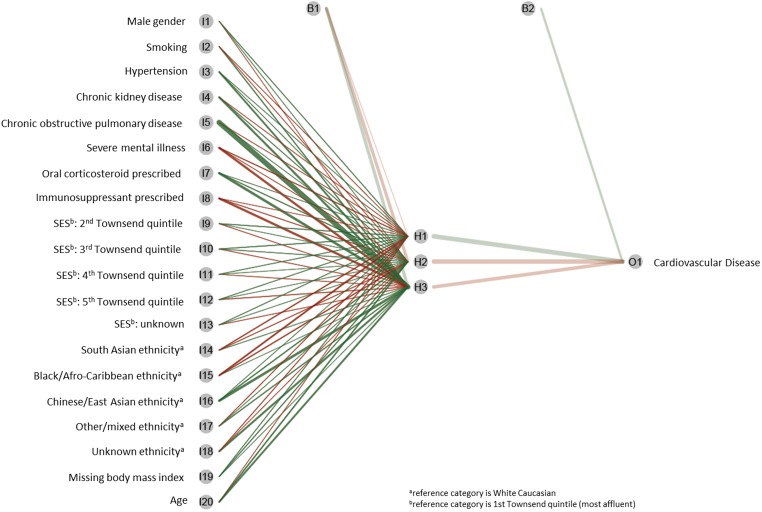
Illuminating “black-box” understanding of machine-learning neural networks: visualization of the risk factors and their association with cardiovascular disease developed from CPRD primary care study population. Green lines are positive predictors, red lines are negative predictors, and the thickness of the line represents the weight (importance) of the risk factor to the outcome.

It is acknowledged that the “black-box” nature of machine-learning algorithms, in particular neural networks, can be difficult to interpret. This refers to the inherent complexity in how the risk factor variables are interacting and their independent effects on the outcome. However, improvements in data visualization methods have improved understanding of these models, illustrating the importance of network connections between risk factors [35] (See example visualising our neural network model in **Fig 2**).

It is also recognised that as the number of potential risk factors increases, the complexity of the models can cause over-fitting, yielding implausible results. We addressed this by active and appropriate choice of pre-training, hyper-parameter selection, and regularisation [36].

Although we have cross-validated the performance of the machine-learning algorithms using an independent dataset, an approach commonly used for the development of established cardiovascular risk algorithms applied to clinical practice [2–5,24,37], it must be acknowledged that the jack-knife procedure may yield more accurate results as demonstrated in genomic or proteomic datasets [38,39]. Moreover, these established risk prediction algorithms for use in clinical practice have been developed from a binary classification framework which can often result in an unbalanced dataset. Ensemble learning have been demonstrated as a solution to construct balanced datasets to enhance prediction performance [40]. These methods are not yet commonplace for developing risk prediction models in clinical datasets but their utility should be explored in future studies.

Finally, we note the study was performed in a large cohort of primary care patients in the UK. However, its demonstration of machine-learning methods, and use of routine clinical data available within electronic records in several countries [41], underline applicability to other populations and health systems.

### Future implications

CVD risk prediction has become increasingly important in clinical decision-making since the introduction of the recent ACC/AHA and similar guidelines internationally [2,42]. Machine-learning approaches offer the exciting prospect of achieving improved and more individualised CVD risk assessment. This may assist the drive towards personalised medicine, by better tailoring risk management to individual patients [43,44].

The improvement in predictive accuracy found in the current study should be further explored using machine learning with other large clinical datasets, in other populations, and in predicting other disease outcomes. Future investigation of the feasibility and acceptability of machine-learning applications in clinical practice will be needed. As the computational capacity in health care systems is improving, the opportunities to exploit machine-learning to enhance prediction of disease risk in clinical practice will become a realistic option [7]. This might increasingly include predicting protein structure and function from genetic sequences from patients’ clinical profiles [7]. This will inevitably require exploration in future studies on utility and clinical applicability other computationally demanding machine-learning algorithms such as support vector machines and deep learning for integration into primary care electronic health records. In several countries, electronic health records across health care organisations are held on central servers. This may allow new algorithm development to be performed off-site using cloud computing software, and then returned to the clinical setting as applications programme interfaces (APIs) for PCs, mobile devices and tablets.

## Conclusion

Compared to an established risk prediction approach, this study has shown machine-learning algorithms are better at predicting the absolute number of cardiovascular disease cases correctly, whilst successfully excluding non-cases. This has been demonstrated in a large and heterogeneous primary care patient population using routinely collected electronic health data.

## Supporting information

S1 Table Full ranking of important variables for four machine-learning 10-year CVD risk prediction algorithms. Variable importance determined based on coefficient effect sizes (logistic regression), frequency (random forest, gradient boosting machines), or weighting (neural networks) developed from the training CPRD training cohort of 295,267 patients.
(DOCX)

S2 Table Classification analysis showing sensitivity, specificity, positive predictive value (PPV), and negative predictive (NPV) value of 10-year CVD machine-learning prediction algorithms. Thresholds are determined corresponding to the ACC/AHA guideline recommendation determining ‘high risk’ > 7.5% for initiating of lipid modification.
(DOCX)

S1 Text Machine-learning algorithms. (DOCX)

## References

[1] World Health Organization. Global Status Report on Noncommunicable Diseases Geneva, Switzerland: World Health Organization, 2014.

[2] Goff DC, Lloyd-Jones DM, Bennett G, Coady S, D’Agostino RB, Gibbons R, et al 2013 ACC/AHA Guideline on the Assessment of Cardiovascular Risk: A Report of the American College of Cardiology/American Heart Association Task Force on Practice Guidelines. Circulation 2013; 135(11): 1–50.

[3] Hippisley-Cox J, Coupland C, Vinogradova Y, Robson J, Minhas R, Sheikh A, et al Predicting cardiovascular risk in England and Wales: prospective derivation and validation of QRISK2. BMJ 2008; 336(7659): 1475–82. 10.1136/bmj.39609.449676.25 18573856 PMC2440904

[4] D’Agostino RB, Vasan RS, Pencina MJ, Wolf PA, Cobain M, Massaro JM, et al General Cardiovascular Risk Profile for Use in Primary Care: The Framingham Heart Study. Circulation 2008; 117(6): 743–53. 10.1161/CIRCULATIONAHA.107.699579 18212285

[5] Ridker P, Buring JE, Rifai N, Cook NR. Development and validation of improved algorithms for the assessment of global cardiovascular risk in women: The reynolds risk score. JAMA 2007; 297(6): 611–9. 10.1001/jama.297.6.611 17299196

[6] Ridker PM, Danielson E, Fonseca FAH, Genest J, Gotto AM, Kastelein JJP, et al Rosuvastatin to Prevent Vascular Events in Men and Women with Elevated C-Reactive Protein. New England Journal of Medicine 2008; 359(21): 2195–207. 10.1056/NEJMoa0807646 18997196

[7] Obermeyer Z, Emanuel EJ. Predicting the Future—Big Data, Machine Learning, and Clinical Medicine. The New England journal of medicine 2016; 375(13): 1216–9. 10.1056/NEJMp1606181 27682033 PMC5070532

[8] Dreiseitl S, Ohno-Machado L. Logistic regression and artificial neural network classification models: a methodology review. Journal of Biomedical Informatics 2002; 35(5–6): 352–9. 12968784 10.1016/s1532-0464(03)00034-0

[9] Berglund E, Lytsy P, Westerling R. Adherence to and beliefs in lipid-lowering medical treatments: A structural equation modeling approach including the necessity-concern framework. Patient Education and Counseling 2013; 91(1): 105–12. 10.1016/j.pec.2012.11.001 23218590

[10] Herrett E, Thomas SL, Schoonen WM, Smeeth L, Hall AJ. Validation and validity of diagnoses in the General Practice Research Database: a systematic review. British journal of clinical pharmacology 2010; 69(1): 4–14. 10.1111/j.1365-2125.2009.03537.x 20078607 PMC2805870

[11] Eeg-Olofsson K, Cederholm J, Nilsson PM, Zethelius B, Svensson AM, Gudbjornsdottir S, et al New aspects of HbA1c as a risk factor for cardiovascular diseases in type 2 diabetes: an observational study from the Swedish National Diabetes Register (NDR). Journal of internal medicine 2010; 268(5): 471–82. 10.1111/j.1365-2796.2010.02265.x 20804517

[12] Emerging Risk Factors Collaboration. C-Reactive Protein, Fibrinogen, and Cardiovascular Disease Prediction. New England Journal of Medicine 2012; 367(14): 1310–20. 10.1056/NEJMoa1107477 23034020 PMC3714101

[13] Jardine AG, Gaston RS, Fellstrom BC, Holdaas H. Prevention of cardiovascular disease in adult recipients of kidney transplants. The Lancet; 378(9800): 1419–27. 10.1016/S0140-6736(11)61334-2 22000138

[14] Mason JE, Starke RD, Van Kirk JE. Gamma-glutamyl transferase: a novel cardiovascular risk biomarker. Preventive cardiology 2010; 13(1): 36–41. 10.1111/j.1751-7141.2009.00054.x 20021625

[15] Mullerova H, Agusti A, Erqou S, Mapel DW. Cardiovascular comorbidity in COPD: systematic literature review. Chest 2013; 144(4): 1163–78. 10.1378/chest.12-2847 23722528

[16] Osborn DP, Hardoon S, Omar RZ, Holt RI, King M, Larsen J, et al Cardiovascular risk prediction models for people with severe mental illness: results from the prediction and management of cardiovascular risk in people with severe mental illnesses (PRIMROSE) research program. JAMA psychiatry 2015; 72(2): 143–51. 10.1001/jamapsychiatry.2014.2133 25536289 PMC4353842

[17] Ray WA, Chung CP, Murray KT, Hall K, Stein CM. Atypical Antipsychotic Drugs and the Risk of Sudden Cardiac Death. New England Journal of Medicine 2009; 360(3): 225–35. 10.1056/NEJMoa0806994 19144938 PMC2713724

[18] Sin DD, Wu L, Man SF. The relationship between reduced lung function and cardiovascular mortality: a population-based study and a systematic review of the literature. Chest 2005; 127(6): 1952–9. 10.1378/chest.127.6.1952 15947307

[19] Souverein PC, Berard A, Van Staa TP, Cooper C, Egberts ACG, Leufkens HGM, et al Use of oral glucocorticoids and risk of cardiovascular and cerebrovascular disease in a population based case–control study. Heart 2004; 90(8): 859–65. 10.1136/hrt.2003.020180 15253953 PMC1768386

[20] Wannamethee SG, Shaper AG, Perry IJ. Serum creatinine concentration and risk of cardiovascular disease: a possible marker for increased risk of stroke. Stroke; a journal of cerebral circulation 1997; 28(3): 557–63. 10.1161/01.str.28.3.557 9056611

[21] Weng SF, Kai J, Guha IN, Qureshi N. The value of aspartate aminotransferase and alanine aminotransferase in cardiovascular disease risk assessment. Open Heart 2015; 2(e000272): 1–10. 10.1136/openhrt-2015-000272 PMC4548065 26322236

[22] Batista GEAPA, Monard MC. An analysis of four missing data treatment methods for supervised learning. Applied Artificial Intelligence 2003; 17(5–6): 519–33.

[23] Bhaskaran K, Forbes HJ, Douglas I, Leon DA, Smeeth L. Representativeness and optimal use of body mass index (BMI) in the UK Clinical Practice Research Datalink (CPRD). BMJ Open 2013; 3(e003389): 1–8. 10.1136/bmjopen-2013-003389 PMC3773634 24038008

[24] Assmann G, Cullen P, Schulte H. Simple Scoring Scheme for Calculating the Risk of Acute Coronary Events Based on the 10-Year Follow-Up of the Prospective Cardiovascular Münster (PROCAM) Study. Circulation 2002; 105(3): 310–5. 11804985 10.1161/hc0302.102575

[25] Hosmer DW, Lemeshow S, Sturdivant RX. Applied Logistic Regression, 3rd Edition New Jersey, USA: John Wiley & Sons; 2013.

[26] Breiman L. Random Forests. Machine Learning 2001; 45(1): 5–32.

[27] Friedman J. Greedy boosting approximation: a gradient boosting machine. The Annals of Statistics 2001; 29(5): 1189–232.

[28] Hagan M, Demuth H, Beale M, De Jesus O. Neural Network Design, 2nd Edition Boston: PWS Publishers; 2014.

[29] Newson R. Comparing the predictive power of survival models using Harrell’s c or Somers’ D. The Stata Journal 2010; 10(3): 339–58.

[30] Newson R. Confidence intervals for rank statistics: Somers’ D and extensions. The Stata Journal 2006; 6(3): 309–34.

[31] The Emerging Risk Factors Collaboration. C-Reactive Protein, Fibrinogen, and Cardiovascular Disease Prediction. New England Journal of Medicine 2012; 367(14): 1310–20. 10.1056/NEJMoa1107477 23034020 PMC3714101

[32] Waljee AK, Higgins PDR, Singal AG. A Primer on Predictive Models. Clinical and Translational Gastroenterology 2014; 5(1): e44. 24384866 10.1038/ctg.2013.19 PMC3912317

[33] Dybowski R, Gant V, Weller P, Chang R. Prediction of outcome in critically ill patients using artificial neural network synthesised by genetic algorithm. The Lancet 1996; 347(9009): 1146–50. 10.1016/s0140-6736(96)90609-1 8609749

[34] Voss R, Cullen P, Schulte H, Assmann G. Prediction of risk of coronary events in middle-aged men in the Prospective Cardiovascular Münster Study (PROCAM) using neural networks. International Journal of Epidemiology 2002; 31(6): 1253–62. 12540731 10.1093/ije/31.6.1253

[35] Olden J, Jackson D. Illuminating the "black box": a randomization approach for understanding variable contributions in artificial neural networks. Ecological Modelling 2002; 2002(154): 135–50.

[36] Bengio Y. Practical Recommendations for Gradient-Based Training of Deep Architectures In: Montavon G, Orr GB, Müller K-R, eds. Neural Networks: Tricks of the Trade: Second Edition Berlin, Heidelberg: Springer Berlin Heidelberg; 2012: 437–78.

[37] Woodward M, Brindle P, Tunstall-Pedoe H. Adding social deprivation and family history to cardiovascular risk assessment: the ASSIGN score from the Scottish Heart Health Extended Cohort (SHHEC). Heart 2007; 93(2): 172–6. 10.1136/hrt.2006.108167 17090561 PMC1861393

[38] Chen J, Long R, Wang XL, Liu B, Chou KC. dRHP-PseRA: detecting remote homology proteins using profile-based pseudo protein sequence and rank aggregation. Sci Rep 2016; 6(32333): 1–7. 27581095 10.1038/srep32333 PMC5007510

[39] Liu B, Long R, Chou KC. iDHS-EL: identifying DNase I hypersensitive sites by fusing three different modes of pseudo nucleotide composition into an ensemble learning framework. Bioinformatics 2016; 32(16): 2411–8. 10.1093/bioinformatics/btw186 27153623

[40] Liu B, Wang S, Dong Q, Li S, Liu X. Identification of DNA-binding proteins by combining auto-cross covariance transformation and ensemble learning. IEEE Trans Nanobioscience 2016; 15(4): 328–44. 28113908 10.1109/TNB.2016.2555951

[41] Kennedy EH, Wiitala WL, Hayward RA, Sussman JB. Improved cardiovascular risk prediction using nonparametric regression and electronic health record data. Medical care 2013; 51(3): 251–8. 10.1097/MLR.0b013e31827da594 23269109 PMC4081533

[42] National Institute for Health and Care Excellence. Cardiovascular disease: risk assessment and reduction, including lipid modification London, UK: National Institute for Health and Care Excellence, 2016. 32200592

[43] NHS England Board. Personalised Medicine Strategy. London, UK: National Health Service England (NHS England), 2015.

[44] Precision Medicine Intiative (PMI) Working Group. The Precision Medicine Initiative Cohort Program—Building a Research Foundation for the 21st Century Medicine. Washington D.C.: National Institutes of Health (NIH), 2015.

