# Supplementary material for: Can machine-learning improve cardiovascular risk prediction using routine clinical data?
Source: PLoS One. 2017 Apr 4;12(4):e0174944. doi: 10.1371/journal.pone.0174944 (PMC5380334; doi:10.1371/journal.pone.0174944)
Supplement: S1 Table — Variable importance determined based on coefficient effect sizes (logistic regression), frequency (random forest, gradient boosting machines), or weighting (neural networks) developed from the training CPRD training cohort of 295,267 patients. (DOCX) [file pone.0174944.s001.docx]

| **Variables** | **Logistic Regression Rank** | **Coefficient** | **Random Forest Rank** | **Frequency** | **Gradient Boosting Machines Rank** | **Frequency** | **Neural Networks Rank** | **Weight** |
| --- | --- | --- | --- | --- | --- | --- | --- | --- |
| Age* | 4 | 0.857 | 1 | 13408872 | 1 | 78358 | 5 | 72.4 |
| BMI | 48 | 0.001 | 9 | 155959 | 13 | 1513 | 43 | 3.8 |
| Systolic blood pressure* | 29 | 0.092 | 13 | 84902 | 9 | 2593 | 29 | 16.3 |
| Total cholesterol* | 31 | 0.077 | 10 | 146018 | 7 | 3035 | 25 | 19.8 |
| HDL cholesterol* | 22 | -0.163 | 5 | 583924 | 5 | 6970 | 28 | 17.6 |
| LDL cholesterol | 44 | 0.011 | 17 | 14263 | 21 | 590 | 46 | 1.9 |
| Triglycerides | 42 | 0.029 | 7 | 224574 | 6 | 3886 | 34 | 8.7 |
| CRP | 43 | 0.017 | 32 | 841 | 26 | 413 | 40 | 4.5 |
| Serum fibrinogen | 45 | 0.010 | 37 | 278 | 31 | 182 | 35 | 7.9 |
| gamma GT | 39 | 0.040 | 18 | 6936 | 17 | 936 | 33 | 8.8 |
| Serum creatinine | 36 | 0.042 | 11 | 124101 | 14 | 1345 | 41 | 4.4 |
| HbA1c | 32 | 0.074 | 6 | 298334 | 8 | 2871 | 44 | 3.6 |
| FEV1 | 47 | -0.007 | 26 | 1831 | 35 | 133 | 47 | 0.5 |
| AST/ALT ratio | 46 | 0.010 | 20 | 5862 | 29 | 267 | 48 | 0.0 |
| Female* | 12 | -0.515 | 2 | 1932619 | 2 | 10861 | 19 | 33.2 |
| Smoking* | 13 | 0.503 | 4 | 1256277 | 4 | 8262 | 18 | 33.2 |
| Family history of CHD < 60 years | 17 | 0.332 | 30 | 915 | 19 | 633 | 23 | 23.5 |
| Ethnicity^a^: South Asian | 3 | 0.978 | 3 | 1623115 | 3 | 9699 | 7 | 64.0 |
| Ethnicity^a^: Black/Afro-Caribbean | 1 | -1.285 | 22 | 3688 | 15 | 1127 | 2 | 97.3 |
| Ethnicity^a^: Chinese/East Asian | 8 | -0.702 | 47 | 0 | 34 | 161 | 4 | 77.7 |
| Ethnicity^a^: Other/Mixed | 2 | -1.008 | 40 | 134 | 30 | 258 | 9 | 50.3 |
| Ethnicity^a^: Unknown | 5 | -0.795 | 48 | 0 | 33 | 166 | 10 | 50.0 |
| SES^b^: 2nd Townsend quintile | 6 | 0.751 | 8 | 178340 | 10 | 2311 | 14 | 46.0 |
| SES^b^: 3rd Townsend quintile | 7 | 0.708 | 15 | 35130 | 11 | 1791 | 11 | 49.6 |
| SES^b^: 4th Townsend quintile | 9 | 0.667 | 16 | 14652 | 18 | 836 | 12 | 48.0 |
| SES^b^: 5th Townsend quintile (most deprived) | 10 | 0.574 | 34 | 557 | 23 | 453 | 16 | 40.1 |
| SES^b^: Unknown | 11 | 0.516 | 29 | 1262 | 32 | 174 | 17 | 38.7 |
| Hypertension | 35 | 0.056 | 33 | 736 | 38 | 96 | 20 | 31.2 |
| Diabetes | 40 | 0.039 | 14 | 71285 | 22 | 454 | 31 | 12.3 |
| Blood pressure treatment* | 28 | 0.092 | 35 | 327 | 40 | 59 | 21 | 30.9 |
| Rheumatoid arthritis | 16 | 0.353 | 42 | 71 | 39 | 96 | 24 | 22.4 |
| Chronic kidney disease | 15 | 0.473 | 45 | 0 | 36 | 118 | 13 | 47.4 |
| Atrial fibrillation | 14 | 0.482 | 23 | 3098 | 25 | 413 | 22 | 25.8 |
| COPD | 18 | 0.300 | 28 | 1370 | 27 | 336 | 1 | 100.0 |
| Severe mental illness | 23 | 0.154 | 46 | 0 | 44 | 37 | 6 | 69.0 |
| Anti-psychotic drug prescribed | 24 | 0.149 | 36 | 289 | 24 | 451 | 32 | 11.2 |
| Oral corticosteroid prescribed | 19 | 0.281 | 21 | 5111 | 20 | 617 | 3 | 78.1 |
| Immunosuppressant prescribed | 27 | 0.093 | 24 | 3050 | 45 | 33 | 8 | 59.2 |
| BMI missing | 25 | -0.131 | 27 | 1629 | 28 | 292 | 15 | 44.4 |
| LDL cholesterol missing | 37 | 0.040 | 39 | 219 | 43 | 37 | 39 | 5.5 |
| Triglycerides missing | 41 | -0.030 | 43 | 40 | 48 | 0 | 45 | 3.6 |
| CRP missing | 34 | -0.058 | 38 | 271 | 41 | 49 | 36 | 7.8 |
| Serum fibrinogen missing | 26 | -0.107 | 41 | 127 | 47 | 10 | 42 | 4.3 |
| gamma GT missing | 33 | -0.064 | 19 | 6217 | 42 | 41 | 38 | 5.6 |
| Serum creatinine missing | 38 | -0.040 | 31 | 877 | 37 | 117 | 37 | 7.3 |
| HbA1c missing | 20 | -0.275 | 12 | 97732 | 12 | 1623 | 27 | 18.0 |
| FEV1 missing | 30 | -0.088 | 44 | 24 | 46 | 22 | 26 | 18.4 |
| AST/ALT ratio missing | 21 | -0.187 | 25 | 1885 | 16 | 967 | 30 | 15.1 |

*core risk factor for ACC/AHA 10-year CVD risk equations

^a^ reference category is White Caucasian

^b^ reference category is 1st Townsend quintile (most affluent)
