# Supplementary material for: Can machine-learning improve cardiovascular risk prediction using routine clinical data?
Source: PLoS One. 2017 Apr 4;12(4):e0174944. doi: 10.1371/journal.pone.0174944 (PMC5380334; doi:10.1371/journal.pone.0174944)
Supplement: S2 Table — Thresholds are determined corresponding to the ACC/AHA guideline recommendation determining ‘high risk’ > 7.5% for initiating of lipid modification. (DOCX) [file pone.0174944.s002.docx]

| **Algorithms** | **Corresponding Thresholds** | **Cases Correct (True Positive)** | **Cases Incorrect (False Negative)** | **Total CVD Cases** | **Non-Cases Correct (True Negative)** | **Non-Cases Incorrect (False Positive)** | **Total Non-Cases** | **Sensitivity (True Positive)** | **Specificity (True Negative)** | **Positive Predictive Value (PPV)** | **Negative Predictive Value (NPV)** |
| --- | --- | --- | --- | --- | --- | --- | --- | --- | --- | --- | --- |
| ACC/AHA Model | 7.5% | 4,643 | 2,761 | 7,404 | 53,106 | 22,479 | 75,585 | 62.7% | 70.3% | 17.1% | 95.1% |
| ML: Random Forest | 71% | 4,834 | 2,570 | 7,404 | 53,297 | 22,288 | 75,585 | 65.3% | 70.5% | 17.8% | 95.4% |
| ML: Logistic Regression | 10% | 4,967 | 2,437 | 7,404 | 53,430 | 22,155 | 75,585 | 67.1% | 70.7% | 18.3% | 95.6% |
| ML: Gradient Boosting Machines | 70% | 4,997 | 2,407 | 7,404 | 53,458 | 22,127 | 75,585 | 67.5% | 70.7% | 18.4% | 95.7% |
| ML: Neural Networks | 32% | 4,998 | 2,406 | 7,404 | 53,461 | 22,124 | 75,585 | 67.5% | 70.7% | 18.4% | 95.7% |
