# Supplementary material for: Can machine-learning improve cardiovascular risk prediction using routine clinical data?
Source: PLoS One. 2017 Apr 4;12(4):e0174944. doi: 10.1371/journal.pone.0174944 (PMC5380334; doi:10.1371/journal.pone.0174944)
Supplement: S1 Text — (DOCX) [file pone.0174944.s003.docx]

**Model Development Process**

To develop the prognostic models, the Clinical Practice Research Datalink (CPRD) observational data were extracted into a labelled dataset containing the independent variables (using the patients’ historical records at their baseline dates) and the dependent variable (occurrence of cardiovascular disease in the 10 years following the baseline date). The labelled dataset in supervised learning was denoted by {**X,**Y} and then partitioned into a training dataset used to develop the model (by learning patterns that are predictive of developing cardiovascular disease within 10 years) and a validation dataset used to internally validate the model.

Prognostic models are generally functions that map the independent variables to a value between 0 and 1 that corresponds to the risk of the dependent variable occurring. Machine-learning aims to learn the function from labelled data. Many machine learning algorithms have hyper-parameters that define a specific function (with parameters) to be learned. For example, random forest has hyper-parameters specifying the number of trees and the max depth of each tree (effectively how many interactions are considered in the model), whereas the decision rules are the parameters. To enable the same data are used to learn the hyper-parameters and the specific function parameters, a process known as cross-validation is often implemented to learn the hyper-parameters while trying to reduce the occurrence of overfitting. Once the optimal hyper-parameters have been learned via cross-validation on the training dataset, the final model parameters are then learned on the whole training dataset and the model is applied to the test set to estimate the performance on new data. In this study we implemented a grid search for the hyper-parameter optimisation. This requires the user to specify a range of values for each hyper-parameters and all possible combinations of the hyper-parameters are investigated, with the combination corresponding to the highest cross-validation performance metric (in this case, maximization of the area under the receiver operating characteristic curve [AUC] being chosen for the final model). The justification for selecting the hyper-parameters that maximises the AUC is that this is less affected when the labelled data are unbalanced compared to using a measure such as accuracy. When the classes are unbalanced, it is also a common strategy to over-sample the rare label data and under-sample the common label data as many machine learning models can be sensitive to unbalanced data. There is an option in *h20* library package in R to perform class balance when training the model, but when we implemented this the performance was not as good, so we left the training of the model to be done on the unbalanced dataset. Below, we describe in further detail the four algorithms used.

**Logistic regression**

The common technique used in medicine for binary outcomes is logistic regression [1]. This is a generalised linear model that models the log odds of the dependant variable (class) as a linear combination of the independent variables (features),

$$\log\left( \frac{p_{i}}{1-p_{i}} \right)=\beta_{0}+\beta_{1}x_{1i}+\ldots+\beta_{m}x_{mi}$$

where the maximum likelihood is used to find the parameters that are most likely given the observed data. It is standard to use a zero mean normal prior for coefficients for regularisation.

**Ensemble learning (random forest & gradient boosting machines)**

The optimal supervised learning model depends on the problem domain. There is no model that consistently outperforms other models for all problems. Consequently, to reduce the variability of models, it is often desirable to combine lots of diverse models. This is known as ensemble learning. There are three population ensembles; bagging, boosting and stacking [2]. Bagging involves creating numerous diverse models by randomly partitioning the training set (i.e., randomly selecting a subset of training examples or randomly selecting a subset of features) and often combining the models using majority voting. An example of a bagging ensemble is random forest [3]. Boosting is another way to combine models, but unlike bagging where the model are created independently, boosting adds models to an existing set of models based on the new model performing well at predicting the outcome for training examples that the existing models in the ensemble performed poorly on. An example of a boosting algorithm are gradient boosting machines [4]. Boosting can reduce the variance and bias of a model [5], but is susceptible to noise [6]. As the class labels in this research have been identified using routine primary care data, there should be little noise in the labels.

**Random forest**

The random forest algorithm [3] averages multiple diverse decision tree predictions. Formally, the algorithm involves learning *ntree* diverse decision trees, $f_{i}\left( x \right):X \to Y$ where **X** is the input variables and Y is the outcome, each trained using a different random selection (with replacement ) of *mtry* variables. The overall prediction then takes the majority voting of all *ntree* decision trees,

$$\left( x \right)= \left\{ \begin{aligned} 1, if\frac{\sum_{i} f_{i}(\boldsymbol{x})}{ntree}>0.5 \\ 0, otherwise \end{aligned} \right.$$

The hyper-parameters of the random forest controls the complexity of the learning function. A random forest with a high max depth (maximum number of interactions between independent variables) and high *mtry* (number of variables included in each tree) is more complex and thus, more likely to cause over-fitting. Therefore, limiting the max depth or *mtry* can effectively perform regularisation and reduce the chance of overfitting. This can also be accomplished by sampling the number of data points to be used for each tree (row sample).

For the hyper-parameter grid search we investigated *ntree* = 50, 100, and 500; *mtry* from 5 up to the maximum number of variables in increments of 5; *max depth* = 2, 4, 6, 8, and 10; row sample of 90%, 95% and 100%. The chosen (optimal) random forest model had the hyper parameters: *ntree* = 500, *mtry* = 35, *max depth* = 6 (up to 6 variable interactions were used by the model) and row sample fraction of 0.95 (95% of the data points were used to train each tree).

**Gradient boosting machines**

Gradient boosting machines [4] aim to minimise the loss function (a measure of difference between the observed and predicted values) by combining a sequence of base-learner models. A common optimisation method to find a minimum is gradient decent which involves going down a gradient to reach a minimum. The key idea behind gradient boosting machines is to sequentially add a new base learner model to the ensemble sequence such that the new model is the model with the greatest correlation with the negative of the loss function’s gradient calculated using the current ensemble sequence predictions.

The gradient boosting machines algorithm [4] is a boosting algorithm that sequentially combines decision trees such that each additional tree is trained with more weighting placed on correctly predicting data-points that the previous decision trees misclassified. In simple terms, each new tree aims to correct for the mistakes of the previous trees. Gradient boosting machines have been successfully implemented across a range of classification tasks but are known to have performance issues when there is noise present in the data.

The hyper-parameters of the gradient boosting machine control the complexity of the learning function. A gradient boosting machine with a high *max depth* (maximum number of interactions between independent variables), high *ntree* (number of trees) and low observations per node (minimum number of data points required for each end node) is more complex and thus, more prone to overfitting. Therefore, limiting the *max depth*, *ntree* or increasing the observations per node can effectively perform regularisation and reduce the chance of overfitting.

The grid search for the hyper-parameters investigated in our models were: *ntree* = 10, 50, 125 and 200; *max depth* = 2, 4, 6, 8, and 10; the minimum observations per node was 2, 10, 50, and 100. The gradient boosting machine model was chosen to have a Bernoulli distribution and the chosen model had the hyper parameters: *ntree* = 125, *max depth* = 4 (up to 4 variable interactions were used by the model) and the minimum number of observations per node was 50.

**Neural network**

A neural network [7] is a supervised learning algorithm consisting of a network with an input layer, hidden layers and output layer. The output of each non-input layer node consists of a weighted linear combination of the previous layer nodes’ outputs transformed by a non-linear function. This is illustrated in the figure below:


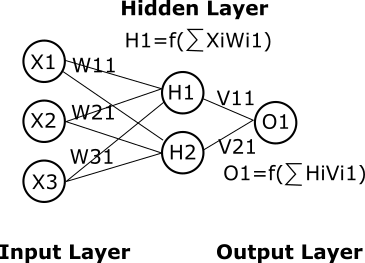


The single hidden layer neural network has been shown to be a universal approximation [8], meaning it is suitable for modelling any function. For the neural networks, the size of the network (number of hidden nodes) controls the complexity of the model whereas the decay performs regularisation. The neural network hyper parameter grid search investigated: *size* = 3,5, 20, 50 and 100; *decay* = 0.00001, 0.001, 0.09, 0.1, 0.5 and 0.9. The chosen optimal neural network model had the hyper parameters: *size* = 3; *decay* = 0.09.

**References**

1. Hosmer DW, Lemeshow S, Sturdivant RX. Applied Logistic Regression, 3rd Edition. New Jersey, USA: John Wiley & Sons; 2013.

2. Valentini G, Masulli F. Ensembles of Learning Machines. In: Marinaro M, Tagliaferri R, eds. Neural Nets: 13th Italian Workshop on Neural Nets, WIRN VIETRI 2002 Vietri sul Mare, Italy, May 30 – June 1, 2002 Revised Papers. Berlin, Heidelberg: Springer Berlin Heidelberg; 2002: 3-20.

3. Breiman L. Random Forests. *Machine Learning* 2001; **45**(1): 5-32.

4. Friedman J. Greedy boosting approximation: a gradient boosting machine. *The Annals of Statistics* 2001; **29**(5): 1189-232.

5. Freund Y, RE S. Experiments with a new boosting algorithm. *ICML* 1996; **96**.

6. Dietterich TG. An Experimental Comparison of Three Methods for Constructing Ensembles of Decision Trees: Bagging, Boosting, and Randomization. *Machine Learning* 2000; **40**(2): 139-57.

7. Hagan M, Demuth H, Beale M, De Jesus O. Neural Network Design, 2nd Edition. Boston: PWS Publishers; 2014.

8. Hornik K, Stinchcombe M, White H. Multilayer feedforward networks are universal approximators. *Neural Networks* 1989; **2**(5): 359-66.
